# Assessment of Carbon Nanoparticle Suspension Lymphography–Guided Distal Gastrectomy for Gastric Cancer

**DOI:** 10.1001/jamanetworkopen.2022.7739

**Published:** 2022-04-18

**Authors:** Yuan Tian, Peigang Yang, Yecheng Lin, Yiyang Hu, Huiyan Deng, Wenqian Ma, Honghai Guo, Yang Liu, Ze Zhang, Pingan Ding, Yong Li, Liqiao Fan, Zhidong Zhang, Dong Wang, Qun Zhao

**Affiliations:** 1 Third Surgery Department, Fourth Hospital of Hebei Medical University, Shijiazhuang, China; 2 Department of Pathology, Fourth Hospital of Hebei Medical University, Shijiazhuang, China; 3 Department of Endoscopy, Fourth Hospital of Hebei Medical University, Shijiazhuang, China

## Abstract

**Importance:**

Carbon nanoparticle suspension injection (CNSI) can be used to visualize lymph node (LN) drainage in gastric cancer. The tracing and diagnostic value of carbon nanoparticle suspension lymphography-guided distal gastrectomy for gastric cancer has not been thoroughly reported.

**Objective:**

To compare the number of lymph nodes identified in patients with gastric cancer receiving a CNSI vs no injection.

**Design, Setting, and Participants:**

This is a retrospective cohort study including patients with clinical T1 to T4 disease who underwent laparoscopic or robotic distal gastrectomy. Data from a cohort of 1225 patients at the Fourth Hospital of Hebei Medical University (Shijiazhuang, China) from November 2019 to February 2021 were analyzed. Patients were divided into the CNSI group and conventional group after 1:1 propensity matching analysis. The mean number of LNs detected was compared between groups, and the diagnostic role of CNSI was analyzed in the CNSI group. Statistical analysis was performed from May to July 2021.

**Exposure:**

CNSI was peritumorally injected under an endoscope 1 day before surgery in the CNSI group, and the conventional group did not receive any treatment before surgery.

**Main Outcomes and Measures:**

The main outcome was the number of LNs detected. Gastrectomy with systematic D1+ (ie, stations 1, 3, 4sb, 4d, 5, 6, and 7) or D2 (ie, all D1 stations, plus 8a, 9, 11p, and 12a) lymphadenectomy was performed. Black-stained LNs and nonblack-stained LNs were examined separately in the CNSI group.

**Results:**

A total of 312 consecutive patients (mean [SD] age, 56.7 [10.4] years; 216 [69.2%] men) who underwent distal gastrectomy were enrolled, including 78 patients in the CNSI group, and another 78 patients determined from 1:1 propensity score matching, making an overall cohort size of 156 patients. The mean (SD) number of LNs detected in the CNSI group was 59.6 (21.4), which was significantly higher than that in the conventional group (30.0 [11.3] LNs; *P* < .001). In the CNSI group, the mean (SD) number of LNs detected at black-stained LN stations was significantly higher than that at nonstained LN stations (9.2 [6.1] LNs per station vs 3.5 [3.2] LNs per station; *P* < .001). For black-stained LN stations, the sensitivity was 97.8% (95% CI, 91.6%-99.6%), specificity was 38.1% (95% CI, 34.2%-42.3%), positive predictive value was 20.1% (95% CI, 16.6%-24.2%), and negative predictive value was 99.1% (95% CI, 96.4%-99.8%); for the black-stained LNs, sensitivity was 97.6% (95% CI, 95.3%-98.8%), specificity was 35.4% (95% CI, 33.9%-36.8%), positive predictive value was 11.6% (95% CI, 10.5%-12.8%), and negative predictive value was 99.4% (95% CI, 98.8%-99.7%).

**Conclusions and Relevance:**

These findings suggest that CNSI was associated with facilitating the dissection of all positive LNs, which could improve surgical quality. Carbon nanoparticle suspension-guided lymphography may be an alternative to conventional systematic lymphadenectomy for distal gastrectomy.

## Key Points

**Question:**

Is use of carbon nanoparticle suspension injection (CNSI) in distal gastrectomy associated with improved detection of lymph nodes (LNs), including metastatic LNs, compared with conventional lymphadenectomy?

**Findings:**

In this cohort study of 312 patients with gastric cancer, significantly more LNs were detected in the CNSI group than in the conventional group. CNSI was associated with accurately identifying metastatic LNs.

**Meaning:**

These findings suggest that CNSI in distal gastrectomy was associated with improved identification of lymph nodes and was diagnostically useful.

## Teaser

This cohort study assesses the ability of carbon nanoparticle suspension injection to detect lymph nodes compared with conventional lymphadenectomy among patients undergoing distal gastrectomy for gastric cancer.

## Introduction

The incidence and mortality of gastric cancer (GC) have decreased; however, it remains one of the most common cancers in the world, ranking as the fifth most commonly diagnosed cancer after lung cancer, breast cancer, colorectal cancer, and prostate cancer and ranking second for mortality.^1,2,3^

Gastrectomy is the only possible cure for advanced GC. Adequate lymph node (LN) dissection plays an important role in gastrectomy, and adequate lymphadenectomy is an important factor for accurate postoperative pathological staging and improved prognosis.^4,5,6^ Unvisualized LNs are difficult to identify in adipose tissue, and standard lymphadenectomy bears the risk of incomplete resection of potentially metastatic LNs.^7^ Moreover, sufficient dissection of LNs from the resected specimen is critical for the evaluation of nodal status.^8,9^

In recent years, various dyes and tracers have been used clinically to observe LN drainage from primary tumors. Carbon nanoparticle suspension injection (CNSI) has been found to provide surgeons with effective visualization of the lymphatic anatomy and sentinel LNs.^10,11,12^

CNSI^13,14^ is different from other tracers and has a diameter of 150 nm. After being injected into the surrounding tissue of the tumor, CNSI is swallowed by macrophages and quickly enters lymphatic vessels but does not enter blood vessels. It is retained in LNs and stains the LNs black, thus achieving the living staining of drainage LNs in the tumor region. In addition, CNSI can be observed in vivo after approximately 3 to 4 months,^13^ allowing sufficient time to observe the stained LNs for adequate intraoperative dissection and postoperative LN detection.

However, the pattern of LN staining of carbon nanoparticles and whether stained lymphography detects all potentially metastatic LNs are unknown. This study aimed to evaluate LN dissection quality and the pattern of LN staining associated with carbon nanoparticle suspension lymphography–guided distal gastrectomy for GC, analyze the diagnostic value associated with peritumoral CNSI injection 1 day before surgery, and analyze the sensitivity associated with CNSI in detecting metastatic LN stations and LNs during distal gastrectomy.

## Methods

This cohort study was approved by the Medical Ethics Committee of the Fourth Hospital of Hebei Medical University, and informed consent was obtained from patients who underwent relevant data analysis. This study is reported following the Strengthening the Reporting of Observational Studies in Epidemiology (STROBE) reporting guideline.

### Patients

We retrospectively reviewed a prospectively collected GC database to identify patients who underwent robotic or laparoscopic gastrectomy between May 2019 and December 2020. The inclusion criteria were age 18 years or older, histologically confirmed adenocarcinoma of the stomach or esophagogastric junction, distal subtotal gastrectomy performed according to the tumor location, and clinical stage T1 to T4a, N0 to N3, and M0. The exclusion criteria were a previous history of other cancers, having received previous curative resection (such as endoscopic submucosal dissection or endoscopic mucosal resection), and having received preoperative chemotherapy or radiation therapy.

### Technique

#### Endoscopic CNSI Injection

**Figure 1.**
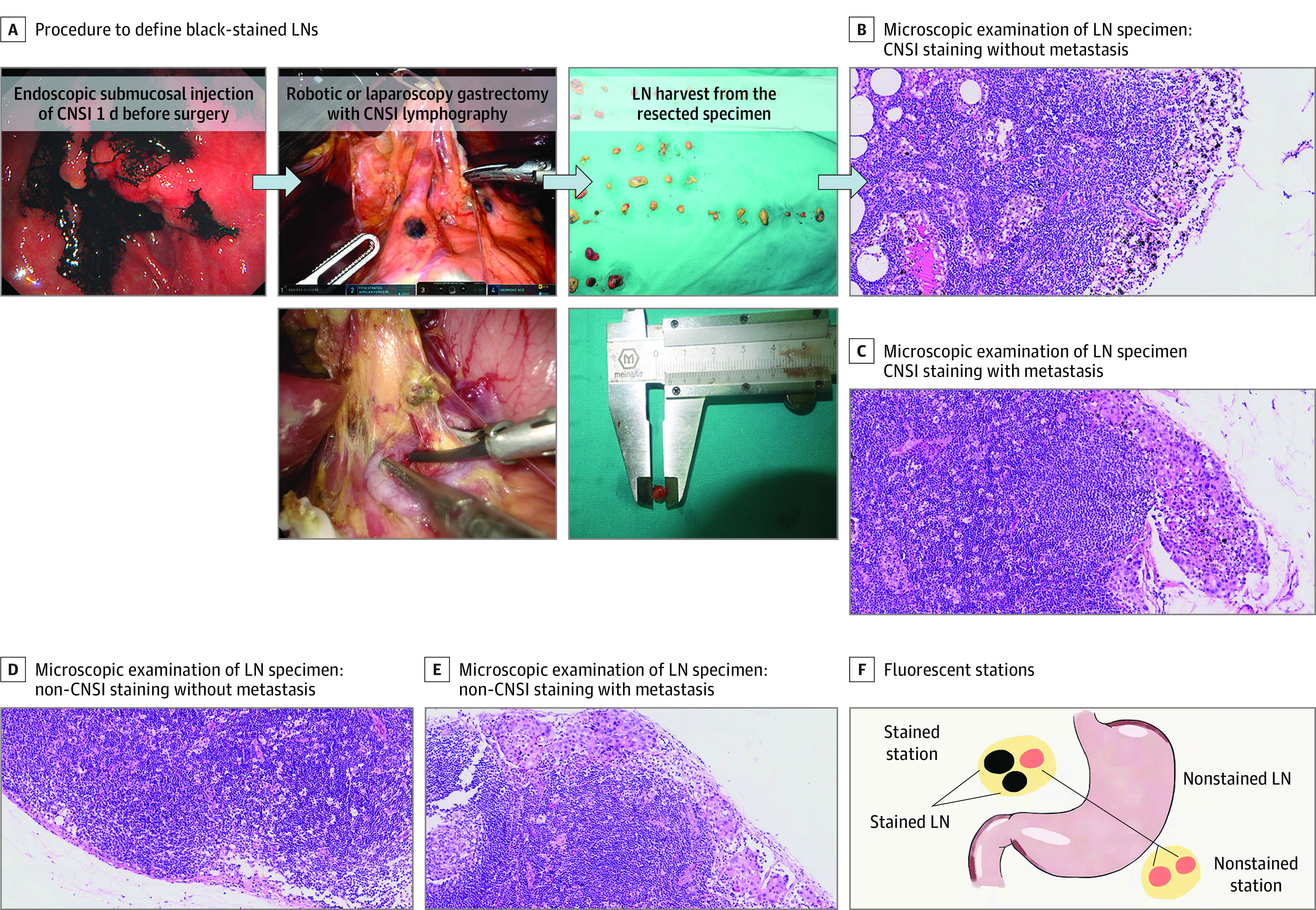
Procedures Performed in the Carbon Nanoparticle Suspension Injection (CNSI) Group and Illustration of Black-Stained Lymph Nodes (LNs) and Stations B-E, hematoxylin and eosin staining, 20× magnification.

CNSI (50 mg per dose, Chongqing Lesmei Pharmaceutical) was marked in the endoscopy division 1 day before surgery. CNSI was injected submucosally at 4 points (proximal side, distal side, and left and right sides) 0.5 to 1 cm from the tumor edge under endoscopy. The test dose for each point was approximately 0.25 mL (Figure 1).

#### Surgery

All patients who were enrolled underwent radical distal subtotal gastrectomy using the da Vinci Surgical Xi system (Intuitive Surgical) or laparoscopy. D1+ (ie, stations 1, 3, 4sb, 4d, 5, 6, and 7) lymphadenectomy was performed for patients with clinically early GC without suspicion of nodal metastases, and D2 (ie, all D1 stations, plus 8a, 9, 11p, and 12a) lymphadenectomy was performed for patients with advanced GC or any suspicion of nodal metastases. When residual stained LNs were identified in the range of D1+ or D2 LN dissection, these LNs were additionally removed (Video; eFigure 1 in the Supplement). Although we occasionally detected stained LNs outside the planned dissection area (eg, stations 4sa, 11d, and 14v), excessive dissection beyond the scope of D2 lymphadenectomy was not performed.

**Video 1.** Carbon Nanoparticle Suspension Injection (CNSI) Tracer-Guided Suprapancreatic Lymph Node Dissection for Gastric Cancer This video illustrates CNSI tracer-guided suprapancreatic lymph node dissection for gastric cancer. CNSI was peritumorally injected under the endoscope 1 day before surgery, and black-stained lymph nodes were seen and completely dissected intraoperatively.

Morbidity and mortality were assessed within 30 days after surgery. Postoperative complications were graded according to the Clavien-Dindo classification.^15^

#### Postoperative LN Harvest

After carbon nanoparticle suspension lymphography–guided lymphadenectomy, each LN station was first separated ex vivo from the resected specimen in accordance with the definitions of the Japanese classification of gastric carcinoma.^16^ LNs were classified as black-stained if they were stained with CNSI and nonstained if they were not stained with CNSI. Stations containing stained LNs were classified as black-stained stations, while those without stained LNs were classified as nonstained stations. Each LN was measured with a Vernier caliper, and LNs smaller than 5 mm were defined as micro-LNs (Figure 1). The presence and absence of CNSI staining were subsequently matched with the results of histopathological analysis. For true-positive (TP) stations, LNs were defined as stained stations and contained metastatic LNs on histopathological examination. For false-negative (FN) stations, LNs were defined as nonstained stations but contained metastatic LNs on histopathological examination. For false-positive (FP) stations, LNs were defined as black-stained but were tumor-free. For true-negative (TN) stations, LNs were defined as nonstained and tumor-free. The sensitivity of carbon nanoparticle suspension lymphography-guided lymphography for detecting LN metastasis in stations or LNs was calculated as *TP* / (*TP* + *FN*). The specificity was calculated as *TN* / (*TN* + *FP*); the PPV was calculated as *TP* / (*TP* + *FP*); and the NPV was calculated as *TN* / (*TN* + *FN*).

### Statistical Analysis

Continuous variables are reported as medians with IQRs, and the *t* test was used to detect differences. Categorical variables are reported as numbers and proportions, and the χ^2^ test or Fisher exact test was used to compare the differences in proportions depending on the application criteria. To overcome biases from the different distributions of covariates among patients in the 2 study groups, a propensity score analysis was performed. The model was used to obtain a 1:1 match using the nearest-neighbor matching method. Patients for whom the propensity score was not applicable were excluded from further analysis. Two-sided *P* < .05 was considered statistically significant. SPSS statistical software version 24 (IBM) was used for all statistical analyses. Statistical analysis was performed from May to July 2021.

## Results

A total of 312 patients (mean [SD] age, 56.7 [10.4] years; 216 [69.2%] men) underwent laparoscopic and robotic distal subtotal gastrectomy from May 2019 to December 2020. Three patients had a previous history of other cancers, 40 patients had received preoperative chemotherapy or chemoradiotherapy, and 12 patients had received previous curative resection. The remaining 257 patients were included in the analysis, of whom 78 received preoperative endoscopic injections of CNSI (CNSI group), and 179 underwent surgery without CNSI (conventional group). After 1:1 propensity matching analysis, there were 78 patients each in the CNSI group and conventional group (eFigure 2 in Supplement). The baseline characteristics of patients in both groups were balanced after matching (Table 1).

**Table 1.** Clinicopathologic Characteristics of the CNSI Group and the Conventional Group After Propensity Score Matching

Characteristic	Patients, No. (%)		*P* value
	CNSI (n = 78)	Control (n = 78)	
Age, mean (SD), y	56.3 (9.7)	57.3 (11.3)	.57
BMI, mean (SD)	25.2 (4.5)	24.2 (5.1)	.86
Sex			
Men	53 (67.9)	48 (61.5)	.40
Women	25 (32.1)	30 (38.5)	
Histologic type (Lauren classification)			
Intestinal	33 (42.3)	33 (42.3)	.54
Diffuse	27 (34.6)	32 (41)	
Mixed	18 (23.1)	13 (16.7)	
Extent of LND			
D1+	14 (17.9)	17 (21.8)	.55
D2	64 (82.1)	61 (78.2)	
Surgical approach			
Laparoscopic	32 (41)	36 (46.2)	.52
Robotic	46 (59)	42 (53.8)	
cT stage			
cT1	18 (23.1)	17 (21.8)	.87
cT2	32 (41)	29 (37.2)	
cT3	15 (19.2)	15 (19.2)	
cT4	13 (16.7)	17 (21.8)	
cN stage			
cN0	42 (53.8)	39 (50)	.77
cN1	11 (14.1)	16 (20.5)	
cN2	19 (24.4)	17 (21.8)	
cN3	6 (7.7)	6 (7.7)	

Abbreviations: BMI, body mass index (calculated as weight in kilograms divided by height in meters squared); CNSI, carbon nanoparticle suspension injection; D1+, stations 1, 3, 4sb, 4d, 5, 6, and 7; D2, all D1 stations, plus 8a, 9, 11p, and 12a; LND, lymph node dissection.

### LN Dissection

A total of 669 LN stations were detected in the CNSI group, including 4647 LNs, and 704 LN stations were detected in the conventional group, including 2343 LNs. A mean (SD) of 59.6 (21.4) LNs per patient were detected in the CNSI group, which was significantly higher than that in the conventional group (30.0 [11.3] LNs per patient; *P* < .001). There was no difference in number of LN stations detected between the CNSI and conventional groups (mean [SD], 8.6 [1.7] LN stations per patient vs 9.0 [2.0] LN stations per patient; *P* = .14).

**Figure 2.**
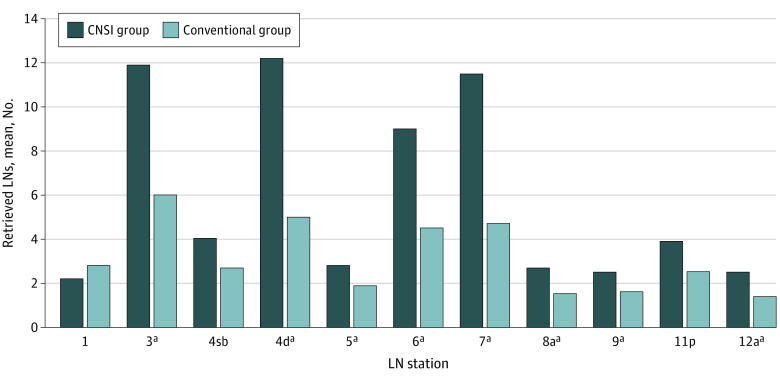
Retrieved Lymph Nodes (LNs) According to LN Station CNSI indicates carbon nanoparticle suspension injection. ^a^*P* < .05 between groups.

**Figure 3.**
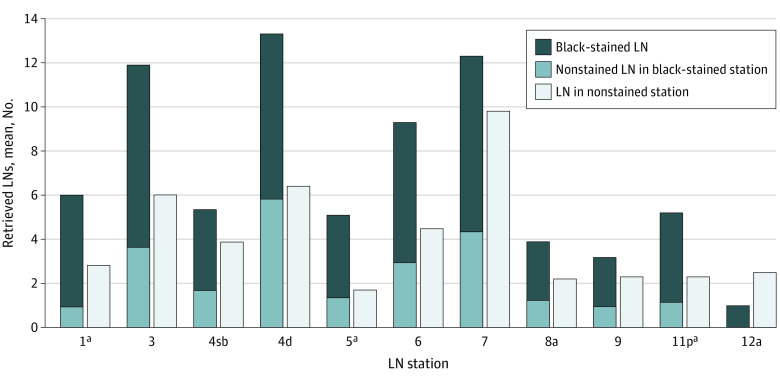
Black-Stained Lymph Nodes (LNs), Nonstained LNs in Black-Stained Stations, and LNs in Nonstained Stations According to LN Station ^a^Black stained LNs and nonstained LNs in black-stained stations vs LNs in nonstained stations, *P* < .05.

A mean (SD) of 6.9 (4.1) LNs per station were detected in the CNSI group, which was higher than the rate in the conventional group (3.3 [2.5] LNs per station). In particular, there were statistically significant differences in stations 3, 4d, 5, 6, 7, 8a, 9, and 12a (Figure 2). In the CNSI group, 447 black-stained stations (66.8%) and 3126 black-stained LNs (67.3%) were detected. There was mean (SD) of 40.1 (16.5) black-stained LNs detected per patient and a mean (SD) of 5.5 (1.4) black-stained stations detected per patient in the CNSI group. Furthermore, in the CNSI group, the mean (SD) number of LNs detected in black-stained stations was significantly higher than that detected in nonstained stations (9.2 [6.1] LNs per station vs 3.5 [3.2] LNs per station; *P* < .001), except station 12a. All LNs detected in station 3 were black-stained, and there were significant differences in the numbers of LNs detected in stained vs unstained stations in station 1 (mean [SD], 5.0 [1.0] LNs vs 1.0 [0.0] LNs; *P* = .002), station 5 (mean [SD], 5.1 [2.1] LNs vs 1.7 [0.5] LNs; *P* = .01), and station 11p (mean [SD], 5.2 [2.7] LNs vs 2.3 [0.9] LNs; *P* = .003) (Figure 3).

The mean (SD) number of LN metastases was not significantly different between the CNSI and conventional groups (4.8 [9.3] LN metastases vs 4.1 [6.8] LN metastases; *P* = .62). The mean (SD) number of micro-LNs in the CNSI group was significantly higher than that in the conventional group (12.5 [8.9] micro-LNs vs 4.5 [3.6] micro-LNs; *P* < .001).

### Diagnostic Value Associated With Carbon Nanoparticle Suspension Lymphography

Except for nonmetastatic LNs in stations 1 and 12a, the metastasis rate of black-stained LNs in other stations was higher than that of nonstained LNs, and there were significant differences in stations 4 sb, 4d, 6, 7, 8a, and 9 (eTable 2 in the Supplement).

Of 78 patients in the CNSI group, 36 (46.2%) had LN metastasis. In 34 of these patients with LN metastases, all metastases were observed in black-stained stations only. In 1 patient with LN metastases, metastases were detected in black-stained and nonstained stations, and 1 patient displayed metastases exclusively in nonstained stations. The sensitivity for the detection of metastatic stations of carbon nanoparticle suspension lymphography was 97.8% (95% CI, 91.6%-99.6%), the specificity was 38.1% (95% CI, 34.2%-42.3%), the positive predictive value (PPV) was 20.1% (95% CI, 16.6%-24.2%), and the negative predictive value (NPV) was 99.1% (95% CI, 96.4%-99.8%). The sensitivity for the detection of metastatic LNs of carbon nanoparticle suspension lymphography was 97.6% (95% CI, 95.3%-98.8%), the specificity was 35.4% (95% CI, 33.9%-36.8%), the PPV was 11.6% (95% CI, 10.5%-12.8%), and the NPV was 99.4% (95% CI, 98.8%-99.7%) (Table 2). This analysis applied only to the CNSI group, and the goal was to determine if black staining could estimate LN metastasis.

**Table 2.** Diagnostic Value Associated With Carbon Nanoparticle Suspension Lymphography for the Detection of Metastatic Stations According to the Total Number of Stations

	Total No.	Metastatic, No.	Nonmetastatic, No.	% (95% CI)			
				Sensitivity	Specificity	PPV	NPV
Stations (n = 669)							
Stained	447	90^a^	357^b^	97.8 (91.6-99.6)	38.1 (34.2-42.3)	20.1 (16.6-24.2)	99.1 (96.4-99.8)
Nonstained	222	2^c^	220^d^				
LNs (n = 4647)							
Stained	3126	363^a^	2763^b^	97.6 (95.3-98.8)	35.4 (33.9-36.8)	11.6 (10.5-12.8)	99.4 (98.8-99.7)
Nonstained	1521	9^c^	1512^d^				

Abbreviations: LN, lymph node; NPV, negative predictive value; PPV, positive predictive value.

^a^ Classified as true-positives.

^b^ Classified as false-positives.

^c^ Classified as false-negatives.

^d^ Classified as true-negatives.

### Postoperative Evaluation

No significant differences between the CNSI and conventional groups were observed in intraoperative blood loss (mean [SD], 55.5 [29.0] mL vs 57.2 [31.0] mL; *P* = .70) and operative time (mean [SD], 180.2 [29.1] minutes vs 184.5 [25.6] minutes; *P* = .33) (eTable 1 in the Supplement). The postoperative recovery process was comparable for both groups. There were no significant differences in time to first flatus, time to ambulation, time to first liquid intake, and postoperative hospital stay between the 2 groups. No significant differences were found between the CNSI and conventional groups regarding the incidence of postoperative complications within 30 days after surgery(6 of 78 patients [7.7%] vs 7 of 78 patients [8.9%]; *P* = .77), nor regarding severity of postoperative complications.

## Discussion

In this cohort study, the total number of LNs detected and the number of LNs detected per station in the CNSI group were significantly higher than in the conventional group, suggesting that carbon nanoparticle suspension lymphography-guided distal gastrectomy achieved sufficient LN detection. In previous GC studies, carbon nanoparticles were used to identify only sentinel LNs in early GC.^9^ The number of LNs detected and the diagnostic value of black-stained LNs have not been discussed, to our knowledge. Compared with India ink, CNSI is relatively safe.^17^ Compared with indocyanine green fluorescence, CNSI does not require complicated surgical procedures and equipment, and making its use simpler than that of indocyanine green fluorescence.^18^ Methylene blue, indigo carmine and other dyes have shorter half-lives than CNSI.^19^ The CNSI black stain contrasts well with the color of adipose tissue; thus, LNs can be easily identified during surgery. The surgeon can achieve en bloc resection of LNs without breakage of lymphatic structures, reducing the risk of intraoperative tumor cell spillage and preventing vascular injury in the surrounding tissue. Moreover, the quality control of lymphadenectomy can be carried out well by determining whether there are residual stained LNs within the scope of the scheduled LN dissection.

In the process of postoperative LN sorting, black-stained LNs are more easily obtained from resected specimens without any other methods,^20^ such as fluorescence laparoscopy, secondary searches, or the Lipodissolve technique. It also was associated with increasing the number of micro-LNs detected compared with conventional treatment. Our results show that with CNSI, the accuracy of negative LNs and LN pathological staging may be improved, and it could provide the guidance for postoperative adjuvant therapy, which in turn may improve the prognosis of patients.^21,22,23,24,25^

The results of this study showed that the number of LNs detected in black-stained stations was greater than that detected in nonblack-stained stations, and the metastasis rate of black-stained LNs was higher than that of nonblack-stained LNs. Both black-stained LNs and stations were highly sensitive to pathological diagnosis, and the NPV of unstained LNs was also satisfactory. Our findings of carbon nanoparticle suspension lymphography–guided distal gastrectomy are not equivalent to those of a 2016 study of sentinel LN dissection.^25^ The purpose of this study was to perform en bloc LN dissection, not only resection of sentinel LNs. A study by Yan et al^26^ using carbon nanoparticles to show sentinel LNs in early GC reported that in black-stained sentinel LNs in early GC, sensitivity was 90%, specificity was 100%, and accuracy was 98.9%. In contrast, the sensitivities of stained LNs or stations in this study to detect metastasis were higher. This result suggests that carbon nanoparticle suspension lymphography–guided distal gastrectomy was associated with a more thorough LN dissection, reduced number of residual positive LNs, and increased number of negative or micro-LNs.

### Limitations

There are some limitations in this study. First, there is no further analysis on the CNSI method and the optimal injection time before the operation for LN detection. Our injection rules were conducted for peritumoral submucosal injection of CNSI 1 day before surgery. Although this timing may result in an increase in additional endoscopy and be painful for patients, it was associated with a reduction in the probability of surgical field pollution compared with subserosal injection. The submucosal injection 1 day before the operation was associated with an increase in the diffusion time of CNSI in the lymph system. Second, this study did not include patients who underwent total gastrectomy, and we will analyze carbon nanoparticle suspension lymphography–guided total gastrectomy for patients with GC in future studies. Third, carbon nanoparticle–guided distal subtotal gastrectomy LN dissection was associated with an increase in the number of LNs detected. However, in this study, owing to the short follow-up time, no long-term survival analysis was performed. We plan provide survival results after further follow-up.

## Conclusions

In this cohort study, carbon nanoparticle suspension lymphography–guided distal gastrectomy was associated with increasing the number of postoperative LNs detected and the accuracy of LN pathological staging. It also was associated with promoting the removal of all potential metastatic LNs, which suggests it may suitable to replace conventional lymphadenectomy for distal gastrectomy.

## Notes

Supplement. **eFigure 1.** Lymph Node Beds During Resection **eFigure 2.** Flowchart of Patient Selection Process **eTable 1.** Surgical Outcome and Morbidity for CNSI and Conventional Groups **eTable 2.** Metastasis Rate of Black-Stained and Nonstained LNs According to LN Station

## References

[1] Bray F, Ferlay J, Soerjomataram I, Siegel RL, Torre LA, Jemal A. Global cancer statistics 2018: GLOBOCAN estimates of incidence and mortality worldwide for 36 cancers in 185 countries. CA Cancer J Clin. 2018;68(6):394-424. doi:10.3322/caac.21492 30207593

[2] Karimi P, Islami F, Anandasabapathy S, Freedman ND, Kamangar F. Gastric cancer: descriptive epidemiology, risk factors, screening, and prevention. Cancer Epidemiol Biomarkers Prev. 2014;23(5):700-713. doi:10.1158/1055-9965.EPI-13-1057 24618998 PMC4019373

[3] Venerito M, Link A, Rokkas T, Malfertheiner P. Gastric cancer—clinical and epidemiological aspects. Helicobacter. 2016;21(suppl 1):39-44. doi:10.1111/hel.12339 27531538

[4] Symeonidis D, Diamantis A, Bompou E, Tepetes K. Current role of lymphadenectomy in gastric cancer surgery. J BUON. 2019;24(5):1761-1767. 31786835

[5] Barreto SG, Sirohi B. Why should we perform a D2 lymphadenectomy in gastric cancer? Future Oncol. 2017;13(23):2009-2012. doi:10.2217/fon-2017-0282 28984466

[6] Songun I, Putter H, Kranenbarg EM, Sasako M, van de Velde CJ. Surgical treatment of gastric cancer: 15-year follow-up results of the randomised nationwide Dutch D1D2 trial. Lancet Oncol. 2010;11(5):439-449. doi:10.1016/S1470-2045(10)70070-X 20409751

[7] Roukos DH, Paraschou P, Lorenz M. Distal gastric cancer and extensive surgery: a new evaluation method based on the study of the status of residual lymph nodes after limited surgery. Ann Surg Oncol. 2000;7(10):719-726. doi:10.1007/s10434-000-0719-0 11129418

[8] Siewert JR, Böttcher K, Stein HJ, Roder JD. Relevant prognostic factors in gastric cancer: ten-year results of the German Gastric Cancer Study. Ann Surg. 1998;228(4):449-461. doi:10.1097/00000658-199810000-00002 9790335 PMC1191515

[9] Wu CW, Hsieh MC, Lo SS, Tsay SH, Lui WY, P’eng FK. Relation of number of positive lymph nodes to the prognosis of patients with primary gastric adenocarcinoma. Gut. 1996;38(4):525-527. doi:10.1136/gut.38.4.525 8707081 PMC1383108

[10] Ya X, Qian W, Huiqing L, . Role of carbon nanoparticle suspension in sentinel lymph node biopsy for early-stage cervical cancer: a prospective study. BJOG. 2021;128(5):890-898. doi:10.1111/1471-0528.16504 32930483

[11] Zhang L, Huang Y, Yang C, . Application of a carbon nanoparticle suspension for sentinel lymph node mapping in patients with early breast cancer: a retrospective cohort study. World J Surg Oncol. 2018;16(1):112. doi:10.1186/s12957-018-1414-6 29914538 PMC6006710

[12] Wang R, Mo S, Liu Q, . The safety and effectiveness of carbon nanoparticles suspension in tracking lymph node metastases of colorectal cancer: a prospective randomized controlled trial. Jpn J Clin Oncol. 2020;50(5):535-542. doi:10.1093/jjco/hyaa011 32083298

[13] Lu Y, Wei JY, Yao DS, Pan ZM, Yao Y. Application of carbon nanoparticles in laparoscopic sentinel lymph node detection in patients with early-stage cervical cancer. PLoS One. 2017;12(9):e0183834. doi:10.1371/journal.pone.0183834 28873443 PMC5584962

[14] Du J, Zhang Y, Ming J, . Evaluation of the tracing effect of carbon nanoparticle and carbon nanoparticle-epirubicin suspension in axillary lymph node dissection for breast cancer treatment. World J Surg Oncol. 2016;14(1):164. doi:10.1186/s12957-016-0925-2 27335011 PMC4918110

[15] Dindo D, Demartines N, Clavien P-A. Classification of surgical complications: a new proposal with evaluation in a cohort of 6336 patients and results of a survey. Ann Surg. 2004;240(2):205-213. doi:10.1097/01.sla.0000133083.54934.ae 15273542 PMC1360123

[16] Japanese Gastric Cancer Association. Japanese gastric cancer treatment guidelines 2018 (5th edition). Gastric Cancer. 2021;24(1):1-21. doi:10.1007/s10120-020-01042-y 32060757 PMC7790804

[17] Hornig D, Kühn H, Stadelmann O, Bötticher R. Phlegmonous gastritis after Indian ink marking. Endoscopy. 1983;15(4):266-269. doi:10.1055/s-2007-1021529 6884285

[18] Tian Y, Lin Y, Guo H, . Safety and efficacy of carbon nanoparticle suspension injection and indocyanine green tracer-guided lymph node dissection during robotic distal gastrectomy in patients with gastric cancer. Surg Endosc. Published online July 12, 2021. doi:10.1007/s00464-021-08630-8 34254184 PMC9001219

[19] Ryu KW, Lee JH, Choi IJ, Bae JM. Preoperative endoscopic clipping: localizing technique of early gastric cancer. J Surg Oncol. 2003;82(1):75-77. doi:10.1002/jso.10191 12501172

[20] Chen QY, Xie JW, Zhong Q, . Safety and efficacy of indocyanine green tracer-guided lymph node dissection during laparoscopic radical gastrectomy in patients with gastric cancer: a randomized clinical trial. JAMA Surg. 2020;155(4):300-311. doi:10.1001/jamasurg.2019.6033 32101269

[21] Hsu JT, Yeh TS, Jan YY. Survival impact of the number of lymph node dissection on stage I-III node-negative gastric cancer. Transl Gastroenterol Hepatol. 2016;1:9. doi:10.21037/tgh.2016.03.02 28138576 PMC5244715

[22] Song W, Yuan Y, Wang L, . The prognostic value of lymph nodes dissection number on survival of patients with lymph node-negative gastric cancer. Gastroenterol Res Pract. 2014;2014:603194. doi:10.1155/2014/603194 24868201 PMC4020362

[23] Biffi R, Botteri E, Cenciarelli S, . Impact on survival of the number of lymph nodes removed in patients with node-negative gastric cancer submitted to extended lymph node dissection. Eur J Surg Oncol. 2011;37(4):305-311. doi:10.1016/j.ejso.2011.01.013 21288685

[24] He H, Shen Z, Wang X, Qin J, Sun Y, Qin X. Survival benefit of greater number of lymph nodes dissection for advanced node-negative gastric cancer patients following radical gastrectomy. Jpn J Clin Oncol. 2016;46(1):63-70. doi:10.1093/jjco/hyv159 26497044

[25] Shoda K, Ichikawa D, Arita T, . Risk stratification according to the total number of factors that meet the indication criteria for radical lymph node dissection in patients with early gastric cancer at risk for lymph node metastasis. Ann Surg Oncol. 2016;23(5)(suppl 5):792-797. doi:10.1245/s10434-016-5553-0 27613556

[26] Yan J, Zheng X, Liu Z, . A multicenter study of using carbon nanoparticles to show sentinel lymph nodes in early gastric cancer. Surg Endosc. 2016;30(4):1294-1300. doi:10.1007/s00464-015-4358-8 26150223

