# Supplementary material for: Assessment of Carbon Nanoparticle Suspension Lymphography–Guided Distal Gastrectomy for Gastric Cancer
Source: JAMA Netw Open. 2022 Apr 18;5(4):e227739. doi: 10.1001/jamanetworkopen.2022.7739 (PMC9016491; doi:10.1001/jamanetworkopen.2022.7739)
Supplement: Supplement. — eFigure 1. Lymph Node Beds During Resection eFigure 2. Flowchart of Patient Selection Process eTable 1. Surgical Outcome and Morbidity for CNSI and Conventional Groups eTable 2. Metastasis Rate of Black-Stained and Nonstained LNs According to LN Station [file jamanetwopen-e227739-s001.pdf]

Supplemental Online Content

Tian Y, Yang P, Lin Y, et al. Assessment of carbon nanoparticle suspension lymphography–guided distal gastrectomy for gastric cancer. *JAMA Netw Open*. 2022;5(4):e227739. doi:10.1001/jamanetworkopen.2022.7739

eFigure 1. Lymph Node Beds During Resection
eFigure 2. Flowchart of Patient Selection Process
eTable 1. Surgical Outcome and Morbidity for CNSI and Conventional Groups
eTable 2. Metastasis Rate of Black-Stained and Nonstained LNs According to LN Station

This supplemental material has been provided by the authors to give readers additional information about their work.

© 2022 Tian Y et al. *JAMA Network Open.*

eFigure 1. Lymph Node Beds During Resection

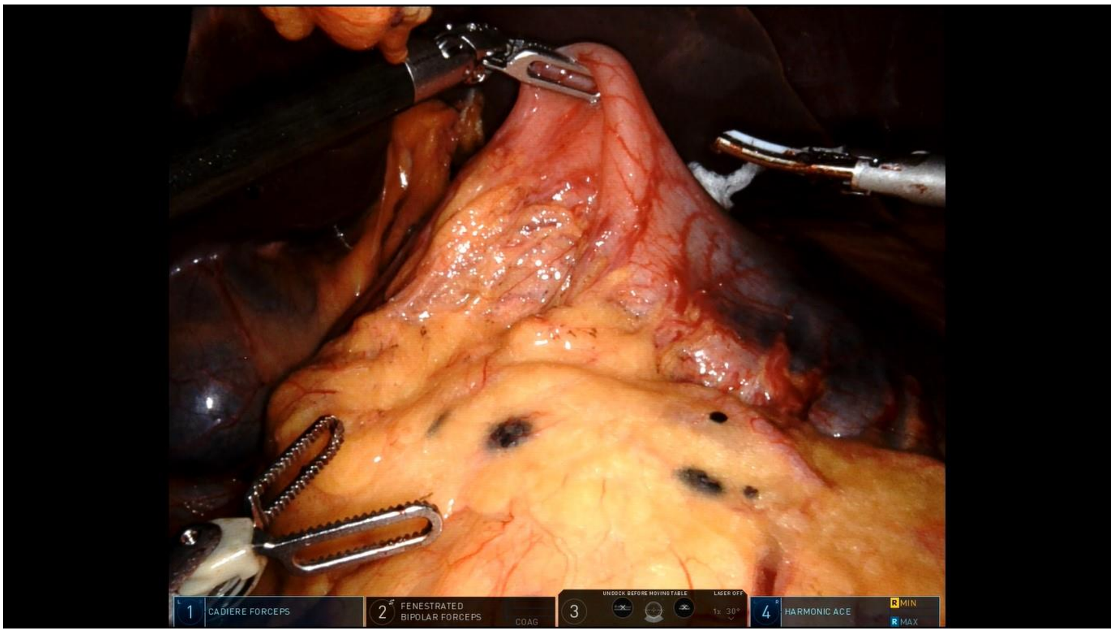

© 2022 Tian Y et al. *JAMA Network Open.*

eFigure 2. Flowchart of Patient Selection Process

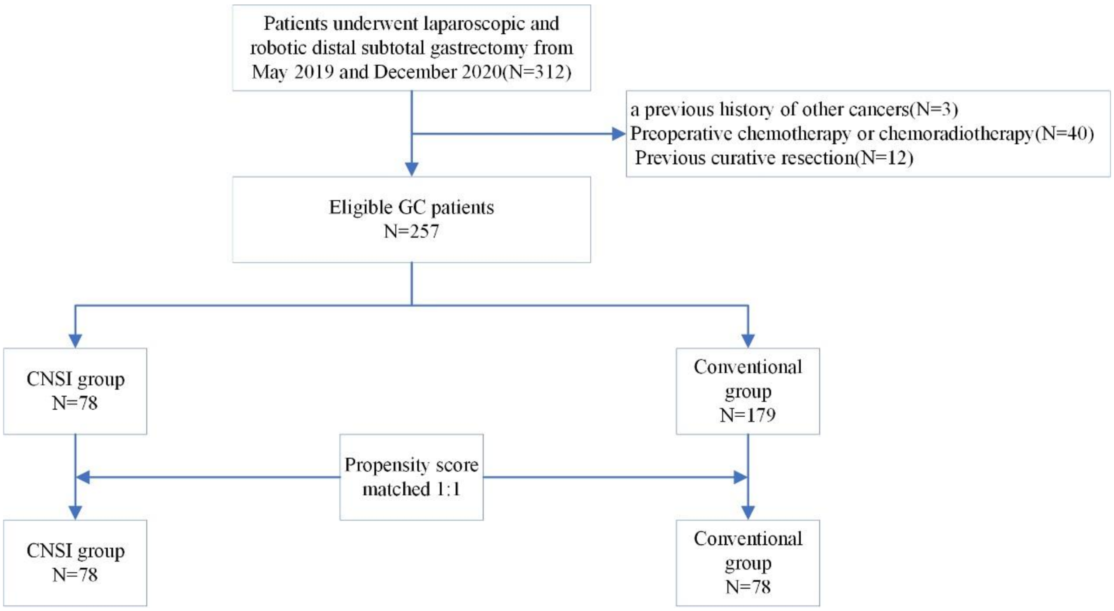

GC, gastric cancer;

CNSI, carbon nanoparticle suspension injection

eTable 1. Surgical Outcome and Morbidity for CNSI and Conventional Groups

	CNSI group (n=78)	Conventional group (n=78)	*P* value
	Mean±SD/N（%）	Mean±SD/N（%）	
**Surgical outcomes**			
Blood loss（mL）	55.4±29.0	57.2±31.0	.70
Surgical time（min）	180.2±29.1	184.5±25.6	.33
First exhaust time(days)	3.1±1.3	3.1±1.5	.96
Postoperative fluid diet intake(days)	5.6±2.0	6.4±3.7	.12
Postoperative hospitalization (days)	8.4±3.1	8.8±3.0	.43
**Morbidity**			
Postoperative complication	6(7.7%)	7(8.9%)	.77
Anastomotic leakage	0（0.0%）	3（3.8%）	.12
Postoperative bleeding	1（1.3%）	1（1.3%）	1.00
Abdominal infection	1（1.3%）	2（2.6%）	1.00
Lymphatic leakage	2（2.6%）	0（0.0%）	.49
Gastroparesis	2（2.6%）	0（0.0%）	.49
Intestinal obstruction	1（1.3%）	0（0.0%）	1.00
Serious pneumonia	1（1.3%）	1（1.3%）	1.00
Cholecystitis	0（0.0%）	1（1.3%）	1.00
**Mortality**	0（0.0%）	0（0.0%）	1.00
**Clavien-Dindo classification**			.53
I	2（2.6%）	1（1.3%）	
II	3（3.8%）	3（3.8%）	
III	1（1.3%）	3（3.8%）	
IV	0（0.0%）	0（0.0%）	
V	0（0.0%）	0（0.0%）	

© 2022 Tian Y et al. *JAMA Network Open.*

eTable 2. Metastasis Rate of Black-Stained and Nonstained LNs According to LN Station

LN station	The metastasis rate of stained LNs[a]	The metastasis rate of non-stained LNs[b]	*P* value
1	0% (0/3)	0% (0/21)	1.00
3	9.9% (21/213)	4.2% (4/96)	.09
4sb	13.8% (4/29)	2.5% (2/80)	.02
4d	19.1% (27/141)	4.1% (6/145)	<0.001
5	10.4% (5/48)	0% (0/30)	.07
6	12.5% (19/152)	3.8% (3/80)	.03
7	10.8% (17/157)	3.7% (5/136)	.02
8a	12.5% (3/24)	0% (0/43)	.02
9	21.4% (3/14)	0% (0/28)	.01
11p	10% (4/40)	0% (0/25)	.10
12a	0% (0/3)	0% (0/57)	1.00

[a] Stained metastatic LNs/stained LNs×100%, [b] Non-stained metastatic LNs/non-stained LNs×100%.

© 2022 Tian Y et al. *JAMA Network Open.*
